# Hats and Baldness

**Published:** 1853-03

**Authors:** 


*Hats and Baldness.* — The subject of baldness is one in which many within the social circle of every medical practitioner are more or less interested; and it is one which probably comes nearer home than this to not a few of our professional readers. On this subject our observations and reflections (Providence be praised! not as yet our *experience*) have led to an abiding conviction. In fact, with the assistance of a scientific friend, we are persuaded that we have made a discovery of considerable practical consequence to those to whom it particularly concerns. The subject is one which may provoke mirth, but a bald pate is often n o joke to the possessor, *capital*

though it be in one sense. He may receive a jocose allusion thereto with an amiable spirit, but there is sadness at the bottom, and his recognition of the jest is usually with a sardonic grin. We state this as a matter of observation, and we feel sure of the correctness of our *diagnosis* in such cases.

What occasions baldness, is a question by no means beneath the dignity of the medical editor. We ask the question with reference to the *male* sex. We do not, as a point of gallantry, presume that women never grow bald; but as a matter of fact they do not nearly as often as do men. This is a fact having an important bearing on our theory. We ask our readers, is it not true that a much smaller proportion of women, than of men, are affected with alopecia? Another *fundamental* (if the reader will excuse the antithesis) fact, viz., *men grow bald almost invariably on the top of the head (alopecia calvities) while the loss of hair, in women, is uniform over the head, or as often laterally, as on the crown.* We put this latter fact in italics, because it is the main spoke in our argument. Now we revert to the first question, what occasions baldness in men? We answer, in imitation of beau Brummel, *Hats is the man.* Seriously, and in the most scientific frame of mind which we are capable of assuming, we believe that male baldness is generally owing to the hat which is commonly worn.

Our train of argument is as follows: Men are affected with baldness much more frequently than women. The baldness in men affects the crown of the head, while in women it is not confined to that situation. There must be adequate reasons for these differences in the two sexes. These reasons, it is rational to conclude, pertain to the articles of dress worn on the head. The hat commonly worn by men differs in some striking peculiarities from the bonnets worn by females. It is logical to infer that the causes of the differences just mentioned, if they relate to the hat, concern the peculiarities in which the hat differs from the bonnet. The legitimacy of this conclusion is sustained, if, on examination of the peculiar features of the hat, it be found that such an effect might *a priori* be expected to occur.

In this discovery we are not entirely original. Such an idea appears lately to have been entertained to some extent, and the hatters, in their latest constructions, have recognized the truth of this idea by making a small ventilating aperture in the crown. This assumes that the objection to the hat lies in the confinement of the air and cutaneous exhalations in the vacant space between the crown of the hat and the crown of the head. There may be something in that supposition. This is one of the peculiarities of the *hat* contrasted with ladies' *bonnets.* The latter permit free ventilation. We do not look upon this feature, however, as the most important in its relation to baldness. The most characteristic trait of the hat is the tightness with which it encircles the head. Herein consists, in our opinion, its agency in the loss of hair. The stove-pipe hat must needs encircle the head tightly, in order to be secure in its position in spite of wind and other disturbing forces. To appreciate the degree of compression, one has only to note the indentation left on the forehead after a tightly fitting hat has been worn for some time. Every body knows how commonly this is to be observed. The head is in fact pretty firmly ligated while the hat is worn. Now what must be the effect of this on the circulation? Plainly, the effect is to interrupt the circulation in the scalp above the circle on which the compression is made. It is precisely like tying a cord around the head sufficiently to diminish, if not stop, for the time, the flow of blood through the temporal and other arteries by which the blood is distributed to the superior portion of the scalp. The hair follicles, as is well known, are very vascular. Their functions require this vascularity, and an adequate, constant supply of oxygenated blood. If this supply be diminished, the growth and nutrition of the hairs are proportionably affected; and, finally, the *pulp* inclosed in the follicle withers and dies, as does any other part when deprived of the *pabulum vitæ.* This effect occurs on the crown because interruption of the circulation in arteries is always felt most in the parts to which the terminal branches are distributed.

Such is our explanation of the fact that baldness is so frequently observed in the young and middle aged men of the present generation. The remedy is to repudiate the present fashion of hats. Let some inventive genius devise a substitute for the unseemly, as well as hair-destructive article which is now the *mode*, and we are firmly convinced that toupees will become objects of curiosity rather than utility, and the bald pate will again be venerated as the distinguishing trait of old age.

We have discharged our duty, in this important matter, by sounding the *tocsin.* It remains for others whose personal interest in the subject is not so remote as ours, to put their shoulders to the wheel, and urge the reform onward.

